# Incremental cost and cost-effectiveness of the addition of indoor residual spraying with pirimiphos-methyl in sub-Saharan Africa versus standard malaria control: results of data collection and analysis in the Next Generation Indoor Residual Sprays (NgenIRS) project, an economic-evaluation

**DOI:** 10.1186/s12936-022-04160-3

**Published:** 2022-06-11

**Authors:** Joshua Yukich, Peder Digre, Sara Scates, Luc Boydens, Emmanuel Obi, Nicky Moran, Allison Belemvire, Mariandrea Chamorro, Benjamin Johns, Keziah L. Malm, Lena Kolyada, Ignatius Williams, Samuel Asiedu, Seydou Fomba, Jules Mihigo, Desire Boko, Baltazar Candrinho, Rodaly Muthoni, Jimmy Opigo, Catherine Maiteki-Sebuguzi, Damian Rutazaana, Josephat Shililu, Asaph Muhanguzi, Kassahun Belay, Joel Kisubi, Joselyn Annet Atuhairwe, Presley Musonda, Nduka Iwuchukwu, John Ngosa, Elizabeth Chizema, Reuben Zulu, Emmanuel Kooma, John Miller, Adam Bennett, Kyra Arnett, Kenzie Tynuv, Christelle Gogue, Joseph Wagman, Jason H. Richardson, Laurence Slutsker, Molly Robertson

**Affiliations:** 1 Tropical Health Consulting LLP, London, UK; 2 PATH, Seattle, WA USA; 3 US President’s Malaria Initiative, US Agency for International Development, Washington, DC USA; 4 PMI VectorLink Project, Abt Associates, Rockville, MD USA; 5 National Malaria Control Programme, Ministry of Health, Accra, Ghana; 6 PMI VectorLink Ghana Project, Abt Associates, Accra, Ghana; 7 AngloGold Ashanti Malaria Control Limited, Accra, Ghana; 8 Programme National de Lutte Contre le Paludisme, Bamako, Mali; 9 US President’s Malaria Initiative, US Agency for International Development, Bamako, Mali; 10 PMI VectorLink Mali Project, Abt Associates, Bamako, Mali; 11 National Malaria Control Programme, Ministry of Health, Maputo, Mozambique; 12 PMI VectorLink Mozambique, Abt Associates, Maputo, Mozambique; 13 National Malaria Control Division, Ministry of Health, Kampala, Uganda; 14 PMI VectorLink Uganda Project, Abt Associates, Kampala, Uganda; 15 US President’s Malaria Initiative, US Agency for International Development, Kampala, Uganda; 16 Uganda Public Health Fellowship Program, Kampala, Uganda; 17 PMI VectorLink Zambia Project, Abt Associates, Lusaka, Zambia; 18 Abt Associates, Lusaka, Zambia; 19 National Malaria Elimination Centre, Lusaka, Zambia; 20 PATH, Lusaka, Zambia; 21 Malaria Elimination Initiative, Global Health Group, University of California San Francisco, San Francisco, CA USA; 22 PATH, Washington, DC USA; 23 IVCC, Washington, DC USA

**Keywords:** Indoor residual spraying, IRS, 3GIRS, NgenIRS, Malaria, Vector control, Cost, Cost-effectiveness, Pirimiphos-methyl, Actellic^®^300CS

## Abstract

**Background:**

Malaria is a major cause of morbidity and mortality globally, especially in sub-Saharan Africa. Widespread resistance to pyrethroids threatens the gains achieved by vector control. To counter resistance to pyrethroids, third-generation indoor residual spraying (3GIRS) products have been developed. This study details the results of a multi-country cost and cost-effectiveness analysis of indoor residual spraying (IRS) programmes using Actellic^®^300CS, a 3GIRS product with pirimiphos-methyl, in sub-Saharan Africa in 2017 added to standard malaria control interventions including insecticide-treated bed nets versus standard malaria control interventions alone.

**Methods:**

An economic evaluation of 3GIRS using Actellic^®^300CS in a broad range of sub-Saharan African settings was conducted using a variety of primary data collection and evidence synthesis methods. Four IRS programmes in Ghana, Mali, Uganda, and Zambia were included in the effectiveness analysis. Cost data come from six IRS programmes: one in each of the four countries where effect was measured plus Mozambique and a separate programme conducted by AngloGold Ashanti Malaria Control in Ghana. Financial and economic costs were quantified and valued. The main indicator for the cost was cost per person targeted. Country-specific case incidence rate ratios (IRRs), estimated by comparing IRS study districts to adjacent non-IRS study districts or facilities, were used to calculate cases averted in each study area. A deterministic analysis and sensitivity analysis were conducted in each of the four countries for which effectiveness evaluations were available. Probabilistic sensitivity analysis was used to generate plausibility bounds around the incremental cost-effectiveness ratio estimates for adding IRS to other standard interventions in each study setting as well as jointly utilizing data on effect and cost across all settings.

**Results:**

Overall, IRRs from each country indicated that adding IRS with Actellic^®^300CS to the local standard intervention package was protective compared to the standard intervention package alone (IRR 0.67, [95% CI 0.50–0.91]). Results indicate that Actellic^®^300CS is expected to be a cost-effective (> 60% probability of being cost-effective in all settings) or highly cost-effective intervention across a range of transmission settings in sub-Saharan Africa.

**Discussion:**

Variations in the incremental costs and cost-effectiveness likely result from several sources including: variation in the sprayed wall surfaces and house size relative to household population, the underlying malaria burden in the communities sprayed, the effectiveness of 3GIRS in different settings, and insecticide price. Programmes should be aware that current recommendations to rotate can mean variation and uncertainty in budgets; programmes should consider this in their insecticide-resistance management strategies.

**Conclusions:**

The optimal combination of 3GIRS delivery with other malaria control interventions will be highly context specific. 3GIRS using Actellic^®^300CS is expected to deliver acceptable value for money in a broad range of sub-Saharan African malaria transmission settings.

**Supplementary Information:**

The online version contains supplementary material available at 10.1186/s12936-022-04160-3.

## Background

Malaria is a major cause of morbidity and mortality globally, especially in Africa, which accounts for 93% of the global malaria burden [1]. Since 2000, substantial reductions in the global burden of malaria have been achieved, due in large part to the large-scale implementation and scale-up of vector control interventions, such as long-lasting insecticidal nets (LLINs) and, in selected areas, indoor residual spraying (IRS) [1, 2]. While LLINs remain the main vector control intervention for malaria in use in sub-Saharan Africa, with over 2 billion cumulatively distributed over the past 20 years, IRS is also a core vector control intervention recommended by the World Health Organization (WHO). IRS has a history of use dating back to the first half of the twentieth century, when dichlorodiphenyltrichloroethane (DDT) and benzene hexachloride were used for large-scale malaria control operations as part of the Global Eradication Programme of Malaria from 1955 to 1969 [3]. Following this period, IRS use was limited in sub-Saharan Africa prior to the start of the US President’s Malaria Initiative (PMI) in 2005. Between 2000 and 2015, IRS was estimated to have contributed to 10% of the total reduction in malaria cases [2]. Despite this success, the proportion of people in sub-Saharan Africa protected by IRS declined between 2010 and 2014. This was likely attributable in part to the switch from IRS products containing pyrethroid insecticides to new, more expensive products with different active ingredients effective at killing pyrethroid-resistant mosquitoes, as well as more targeted approaches to IRS implementation in many countries [1].

Resistance to pyrethroids, the class of insecticides most used for LLINs and until recently also commonly used for IRS, is widespread [1, 4, 5]. As a result, third-generation indoor residual spraying (3GIRS) products that are designed to be effective at killing pyrethroid-resistant mosquitoes for at least six months have been developed [6]. The first 3GIRS products recommended by the WHO for malaria vector control include Actellic^®^300CS, a microencapsulated formulation of pirimiphos-methyl, an organophosphate insecticide; SumiShield^®^ 50WG, which is based on clothianidin, the first neonicotinoid insecticide intended for public health applications; and Fludora^®^ Fusion, a combination product utilizing a mixture of clothianidin and a pyrethroid, deltamethrin. Until recently, there has been limited evidence of the effectiveness of IRS programmes using 3GIRS products and little information available on their cost and cost-effectiveness in deployment at scale. The Next Generation IRS (NgenIRS) project was a 4-year (2016–2019) market-shaping and evidence strengthening effort for 3GIRS products under which the studies described in this manuscript were conducted [6].

A systematic review of the costs of malaria control interventions found that IRS, while being more expensive than LLIN interventions, was consistently estimated to meet WHO criteria for being considered a cost-effective intervention in low-income settings [7]. However, the review identified no cost or cost-effectiveness studies published after the year 2001, and thus did not include the more recent expansion of IRS geographies and products in sub-Saharan Africa. A more recent systematic review covering literature from 2005 to 2018 found no new cost-effectiveness studies of IRS published in this period [8].

This study details the results of a multi-country cost and cost-effectiveness analysis of IRS programmes using Actellic^®^300CS in sub-Saharan Africa in 2017. The study was designed to establish if 3GIRS using Actellic^®^300CS can yield acceptable value for money when applied in addition to standard of care malaria control programmes in a wide range of sub-Saharan African contexts. In addition, this study provides new evidence on the cost and cost-effectiveness of IRS as no new estimates of IRS cost-effectiveness have been published in more than 20 years. The data on cost across varied settings may prove useful for programme decision-making and as inputs to future modelling studies and economic evaluations of decision analytic studies.

## Methods

### Programme selection

Six independent 3GIRS programmes using Actellic^®^300CS across five countries (Ghana, Mali, Mozambique, Uganda, and Zambia) are included in this analysis. Cost data from IRS implementation for all programmes and effect estimates for PMI-funded programmes in Ghana, Mali, Uganda, and Zambia are presented. These programmes were selected for evaluation based on the availability of routine data, documented insecticide-resistance profiles, and whether they had secured funding for IRS implementation. Consideration was also given to representing geographical and vector species diversity (such as both *Anopheles funestus* and *Anopheles gambiae*). Table 1 provides programmatic indicators from each IRS programme. Detailed programme descriptions are included in Additional file 1.

**Table 1:** Description of programme years included in effect estimates and cost analyses

Programme	Year	Number of districts	Approximate annual incidence	Pyrethroid resistance status of primary vector by WHO bioassay	LLIN coverage (%)	LLIN use (%)	Insecticide product	Target dose	Expected m^2^ per structure	Structures sprayed	Expected persons per structure	Persons protected^a^
Ghana AIRS/VectorLink *Northeast and Northern Regions*	2017^b^	7	High (~ 200 per 1000 person-years)	Highly resistant (0–80% mortality in *An. gambiae*)	> 80	50–60	Actellic^®^ 300CS	1 g/m^2^	54.4	304,648	2.7	840,438
	2018	7								277,530		777,475
Ghana AGAMal *Upper West, Upper East Regions*	2017	13	High (~ 300 per 1000 person-years)	Highly resistant (10–85% mortality in *An. gambiae*)	> 90	54–63	Actellic^®^ 300CS	1 g/m^2^	40.0	915,140	1.1	1,028,523
	2018	10								525,377	1.1	646,534
Mali AIRS/VectorLink *Mopti Region*	2017^b^	4	High (~ 300 per 1000 person-years)	Highly resistant (6–90% mortality in *An. gambiae*)	85–90	67–78	Actellic^®^ 300CS	1 g/m^2^	90.0	227,646	3.6	823,201
Mozambique AIRS/VectorLink *Zambezia Province*	2017	7	High (~ 400 per 1000 person-years)	Variable resistance (7–86% mortality in *An. funestus*)	> 90	85–90	Actellic^®^ 300CS	1 g/m^2^	132.0	381,533	3.9	1,933,484
	2018	4							120.0	237,194	4.4	1,121,543
Uganda Abt bilateral *Northern and Eastern Regions*	2017^b^	14	High (~ 200 per 1000 person-years)	Highly resistant (0–80% mortality in *An. gambiae*)	90	74	Actellic^®^ 300CS	1 g/m^2^	101.0	1,225,644	3.5	4,227,236
	2018	15								1,292,309		4,436,156
Zambia AIRS/VectorLink *Eastern, Luapula, Muchinga, Northern Provinces*	2017^b^	4	High (~ 200 per 1000 person-years)	Resistant (40–100% mortality in *An. gambiae*)	55–80	40–60	Actellic^®^ 300CS	1 g/m^2^	66.5	634,371	4.7	3,005,676

AGAMal, AngloGold Ashanti Malaria Control; AIRS, Africa Indoor Residual Spraying Project; CS, capsule suspension; g, gram; LLIN, long-lasting insecticidal net; m, metre; WHO, World Health Organization

^a^Persons protected, as collected during programme implementation, refers to the total number of residents living in houses that were sprayed

^b^Costs from Abt programmes were calculated from the 2017 spray campaigns

### Programme description

Detailed descriptions of each programme were developed using key informant interviews and reviews of programme documents. These descriptions guided the methods used to collect cost data (detailed below).

### Effectiveness study data sources and analysis

The effectiveness of 3GIRS using Actellic^®^300CS in each site was established, as described previously [9–14], using passive malaria case surveillance data reported in national health information systems. In brief, population-based malaria case incidence rates were calculated using health catchment and/or district population estimates. Cases represented patients with suspected malaria who had sought care in the public health system and had received a confirmatory diagnosis, either with a positive malaria rapid diagnostic test result or by microscopy. To describe the seasonal impact of IRS, the cumulative incidence of rapid diagnostic test-positive malaria cases observed during the 6 months following the completion of each IRS campaign was calculated for the IRS districts and compared to the cumulative malaria incidence observed in neighbouring, non-IRS comparator districts with similar current LLIN coverage and historically similar transmission patterns during the same months. In Zambia, the effect estimate was calculated using data across a full year [14]. Cases averted were estimated by applying non-IRS district incidence rates to the populations of the IRS districts to estimate an expected number of cases, then subtracting the observed number of cases. The incidence rate ratios (IRRs) with uncertainty bounds were calculated by comparing health facility–confirmed case incidence in districts or health facilities with IRS to health facility–confirmed case incidence in districts or facilities without IRS during the 6-month (or 1 year) post-spray analysis window, the expected duration of indoor residual efficacy for 3GIRS products. All districts included in the analysis benefitted, at a minimum, from universal bed net distribution campaigns designed to achieve universal coverage of the population at risk with pyrethroid-only LLINs, access to prompt and effective case management, and intermittent preventive treatment in pregnancy. Additionally, seasonal malaria chemoprevention was implemented in Mali in both 3GIRS and comparator districts.

To make the most use of the data available and to provide a single parameter to inform a generalized cost-effectiveness sensitivity analysis, a meta-analysis of effect estimates across country studies was conducted. The analysis used both a ‘fixed effects’ approach as well as a DerSimonian–Laird random-effects model meta-analytic approach, both of which were implemented using the “metafor” package (version 2.1-0) in R (version 3.6.2) [15]. Results were presented in a forest plot, providing a pooled effect estimates of 3GIRS as well as testing for heterogeneity between study sites.

### Cost collection and analysis

Cost data were collected from programmes in Ghana, Mali, Mozambique, Uganda, and Zambia. Cost data collection used a bottom-up approach, meaning that where possible, an ingredients approach was utilized, and the price and quantity of all inputs were estimated. Where this information was not available, line item aggregated expenditures from the IRS programmes were utilized directly. The cost analysis takes the provider perspective and estimates the gross cost of 3GIRS implementation with Actellic^®^300CS.

Both financial costs (expenditures attributable to the provider of the intervention) and economic costs (a measure of the resources used by the provider of the intervention representing the opportunity costs of those resources) were quantified and valued. Capital items included wholly owned vehicles, warehouses, offices, and spray pumps. Costs for capital goods (those with useful lifetimes longer than 1 year) were either treated by using a project-based lifetime or by annuitizing them with an item-specific lifetime. All annuitization assumed a 3% discount rate. Commodities were necessary supplies, such as paper, printer cartridges, personal protective equipment, clothing, and spray pump repair parts. Domestic contributions refer to capital and recurrent costs financed or provided by local government rather than by international donors. Insecticide was the full recurrent cost of the insecticide used during the spray campaign. Local administration consisted of personnel costs for administration and supervision incurred in-country by the implementing agencies, but not including domestic government contributions. Local labour included the personnel costs of spray teams, field supervisors, and other personnel directly involved in the spraying process. Spray operations were other recurrent costs such as vehicle rental, fuel, and maintenance necessary for the implementation of spray campaigns. Finally, international labour consisted of the costs of international technical assistance and personnel not stationed in the spray countries as well as international grant administrators directly applicable to the spray operations.

All costs were converted to 2017 US dollars (USD) by first converting them from the recorded currency to USD using an annual average exchange rate for the period in which the cost was incurred and then inflating them to 2017 USD, where necessary, using the US gross domestic product (GDP) deflator [16, 17]. These inputs comprised a total cost for one annual set of spray rounds.

Cost data collection consisted of review and validation of existing financial and operational reports, direct interviews with programme managers, and review of receipts and invoices. In many cases, a large proportion of support for implementation was provided through PMI and implemented by Abt Associates through global funding mechanisms such as the Africa Indoor Residual Spraying (AIRS) Project/VectorLink. Support for implementation was also provided through bilateral funding agreements in some locations. In some cases, resource use and expenditure data were also collected from the Global Fund to Fight AIDS, Tuberculosis and Malaria and private-sector entities—AngloGold Ashanti Malaria Control Ltd. (AGAMal), for example, in Ghana.

As part of the PMI VectorLink project implementation, Abt Associates produces a cost analysis of all country IRS programmes. These reports formed the first foundation of cost data collection for PMI-supported IRS programmes. Additional data collection was undertaken to identify domestically financed contributions, as Abt Associates reports generally only included costs derived from direct Abt Associates activities, and thus may have excluded substantial in-kind or monetary contributions from other sources. Household costs were not included, nor were potential cost savings from malaria cases averted.

To derive unified information and uncertainty around cost, a meta-analytic approach was used to estimate unit costs by calculating both the mean and standard deviation of the measured per person targeted unit cost from each included programme, since each programme costing represented an independent study of the costs of 3GIRS using Actellic^®^300CS. The parameters of a log-normal distribution that captured the uncertainty in unit costs for Monte Carlo simulation were estimated using the “MASS” package in R [18].

### Outcomes

The main indicator of interest for the costing exercise was cost per person targeted. This indicator incorporates the risk reduction for those living in communities with high coverage (even if one’s house was not sprayed), which arises due to the community effect of IRS. The cost per person protected was the common alternative indicator, but this only considers people living in sprayed houses. For programmes where the number of persons targeted was unavailable in relation to the total cost estimates for the programme, the cost per person targeted was calculated by adjusting the population size up or the unit cost down according to this relationship:

$$\frac{Total\;cost*Program\;coverage}{{Total\;number\;of\;persons\;protected}} = \frac{Total\;cost}{{Total\;number\;of\;persons\;targeted}}$$

The adjustment was necessary as the effect estimates were measured among the entire population targeted, rather than only the population protected (living in sprayed houses). Using the higher cost per person protected would result in a conservative bias in the estimation of average and incremental cost-effectiveness ratios. In the cases where reported programme coverage was greater than 100%, no adjustment was made. Where no coverage data were available, 80% coverage was assumed.

### Estimation of impact and cost-effectiveness

The IRRs were used to calculate an estimated number of cases averted in each of the IRS study areas in relation to non-IRS study areas. Cases averted were used to calculate deaths averted assuming a case fatality rate among malaria cases (0.2%) [19], and that all malaria deaths occurred in children, and that there were 33 disability-adjusted life years (DALYs) per death [1, 20, 21]. This simplifying assumption includes age-weighting and a 3% discount rate. Removing age weighting would result in minimal changes (~ 31 years of life lost per death). Removal of time discounting would result in a substantial increase in the absolute numbers of DALYs averted because most malaria deaths occur during early childhood when life expectancy is near its peak. As time discounting is conservative for incremental cost-effectiveness ratio (ICER) estimation, it is included here. An ICER was calculated for each case, death, and DALY averted based on programme-specific costs.

3GIRS using Actellic^®^300CS was determined to be highly cost-effective or cost-effective if the cost per DALY averted was equivalent to the 2018 GDP per capita or three times the GDP per capita, respectively, per WHO standard thresholds. In addition, a 0.5 times GDP per capita threshold, which was termed the stringent highly cost-effective threshold, was included [22, 23]. The Consolidated Health Economic Evaluation Reporting Standards (CHEERS) guidelines and the Gates Reference Case were followed in collection analysis and presentation of the cost-effectiveness results [24, 25].

### Sensitivity analyses

A probabilistic sensitivity analysis was used to generate plausibility bounds around the ICER estimates for adding 3GIRS using Actellic^®^300CS to other standard interventions. The probabilistic sensitivity analysis was conducted for each study setting. The ICER outcomes were simulated using Monte Carlo methods, assuming no covariance between unit costs and effects for a population of 100,000 persons. Ten-thousand simulations were conducted for each country simulation, with an assumption that the cost of the spraying was as measured in each programme per person targeted, with a standard deviation of 1.43 USD in each programme. Since per programme cost estimates give only one specific number, the standard deviation of expected cost was estimated using all available data, and the mean cost was fixed to the point estimate for the programme and modelled using a log-normal distribution. The IRR effects were modelled using log-normal distribution, and a standard deviation was derived from programme-specific estimates. The baseline incidence was based on the World Malaria Report 2018 reported incidence, estimated nationwide for each specific programme. In addition, a locally specific baseline incidence derived from the comparison districts for each country effectiveness analysis was used. Since IRRs estimating the impact of IRS were calculated using only the 6 months of surveillance data following the application of IRS, the estimated effects were applied to only 75% of the baseline annual incidence, as approximately 75% of the annual burden of malaria occurred during the 6-month post-IRS window in all study locations except Zambia where the IRR was calculated using a data from the full year (see Additional file 2: Table S1). Model parameters are presented in Table 2.

**Table 2:** Probabilistic sensitivity analysis parameters

Programme	Baseline incidence (cases per person per year)	Cost estimate (USD)	Standard deviation of cost	Effect estimate (IRR)	Standard deviation of effect (on log scale)
Ghana AIRS/VectorLink *Northeastern and Northern Regions*	0.39	5.21	1.43	0.60	0.11
Mali AIRS/VectorLink *Mopti Region*	0.26	7.76	1.43	0.68	0.20
Uganda Abt bilateral *Northern and Eastern Regions*	0.20	5.53	1.43	0.53	0.20
Zambia AIRS/VectorLink *Eastern, Luapula, Muchinga, Northern Provinces*	0.21	3.35	1.43	0.88	0.20
Global sensitivity analysis	Varied (from 0.001 to 1 per person-year)	5.33	1.43	0.67	0.15

Additional parameters	Parameter	Point estimate	Distribution	Sensitivity	Distribution
Case fatality rate	Proportion of incident cases resulting in death	0.002	Fixed	Reduced by 50% to 0.001 in global probabilistic sensitivity analysis	N/A
DALYs per death	Estimated number of DALYs accrued per each death	33	Fixed	Not analysed	N/A

AIRS, Africa Indoor Residual Spraying Project; DALY, disability-adjusted life year; IRR, incidence rate ratio; N/A, not applicable; USD, US dollars

A global probabilistic sensitivity analysis was used to generate plausibility bounds around the ICER estimates for adding 3GIRS using Actellic^®^300CS to other standard interventions. For each baseline incidence rate (per person-year) 1000 Monte Carlo simulations of cost, assuming a log-normal distribution of cost with a mean on the normal true scale of 5.33 USD and a standard deviation of 1.43 USD, the effectiveness of 3GIRS using Actellic^®^300CS was assumed to have an IRR of 0.67 with a 95% confidence interval (CI) ranging from 0.50 to 0.91 (both cost and effect parameters were derived from the meta-analyses presented here). Additionally, 1000 simulations of expected effect size were also conducted for each baseline incidence (from 10 per 1000 person years to 1000 per 1000 person years) and at two levels of case fatality rate (CFR) including a CFR of half that used in the base case scenarios. The simulation results were used to calculate an ICER for each simulation in terms of total cost per DALY averted in a population of 100,000 over the course of 1 year, and these were summarized as the median and 95% quantiles.

One-way sensitivity analyses were conducted to estimate the incremental cost of 3GIRS using Actellic^®^300CS compared to IRS using alternative products at a lower cost. Costs for alternative active ingredients were estimated based on input from IVCC and reflected recent market transactions.

## Results

### Effectiveness

Overall, IRRs from each country indicated that adding 3GIRS using Actellic^®^300CS to the local standard intervention package was expected to be protective compared to the standard intervention package alone. The results of the effectiveness studies are summarized in Table 3. Effect estimates varied across settings, with the most effective IRR arising in Ghana, under the AIRS/VectorLink programme, though the 95% CI around this estimate was wide.

**Table 3:** Effectiveness estimates from the NgenIRS project

Programme	Years(s)	Incidence rate ratio estimate	Lower 95% CI	Upper 95% CI	Estimated cases averted	Estimated persons targeted^a^
Ghana AIRS/VectorLink *Northeastern and Northern Regions*	2015–2017	0.60	0.36	1.00	257,162	597,895
Mali AIRS/VectorLink *Ségou Region*	2015–2016	0.68	0.52	0.89	349,688	304,654
Uganda Abt bilateral *Northern and Eastern Regions*	2016	0.53	0.43	0.66	245,331	1.78 million
Zambia AIRS/VectorLink *Eastern, Luapula, Muchinga, Northern Provinces*	2017	0.88	0.82	0.95	N/A^b^	N/A

Effectiveness results were produced by separate efforts

AIRS, Africa Indoor Residual Spraying Project; CI, confidence interval; N/A, not applicable

^a^The total population of districts that received an IRS intervention during the analysis timeframe

^b^To calculate cases averted by 3GIRS, it was assumed that baseline incidence aligned with the World Malaria Report 2018

**Fig. 1:**
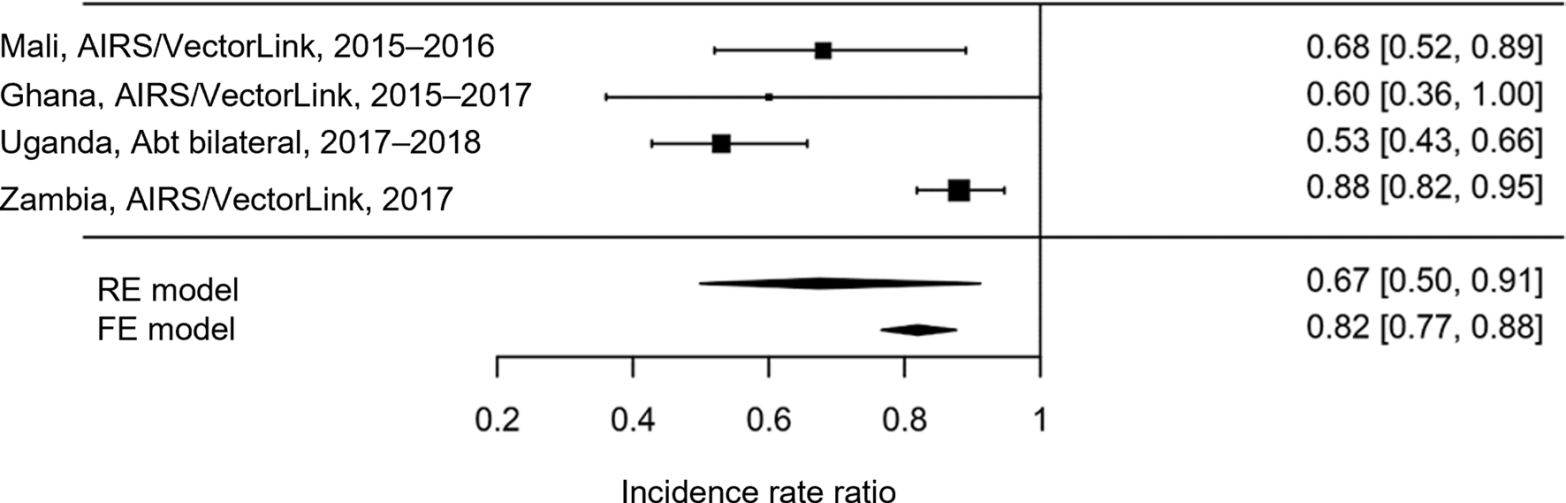
Meta-analysis of effect estimates of IRS versus no IRS from observational studies in NgenIRS countries. AIRS, Africa Indoor Residual Spraying Project; IRS, indoor residual spraying; NgenIRS, Next Generation Indoor Residual Sprays project; RE, random effects; FE, fixed effects

Figure 1 shows the study and meta-analysis results as a forest plot: the summary estimate of the effect of 3GIRS using Actellic^®^300CS from the random-effects model was an IRR of 0.67 (95% CI 0.50–0.91), indicating that 3GIRS was associated with an approximately 33% reduction in malaria case incidence when added to other standard interventions. This was more effective but also more variable than the effect estimate derived from the fixed effect model (IRR 0.82 [95% CI 0.77–0.88]). There was statistically significant heterogeneity between studies (I^2^ = 86.83%; test for heterogeneity: *Q*(df = 3) = 22.7825, *p* < 0.0001) though all studies resulted in significant protective effects for 3GIRS using Actellic^®^300CS. Therefore the study site results were pooled using the DerSimonian–Laird random-effects model and the heterogeneity incorporated into probabilistic sensitivity analysis through the overall pooled effect and variance estimates. In addition, heterogeneity of effect was incorporated into site-specific probabilistic sensitivity analysis using the site-specific IRRs and their associated standard error estimates.

### Costs

**Fig. 2:**
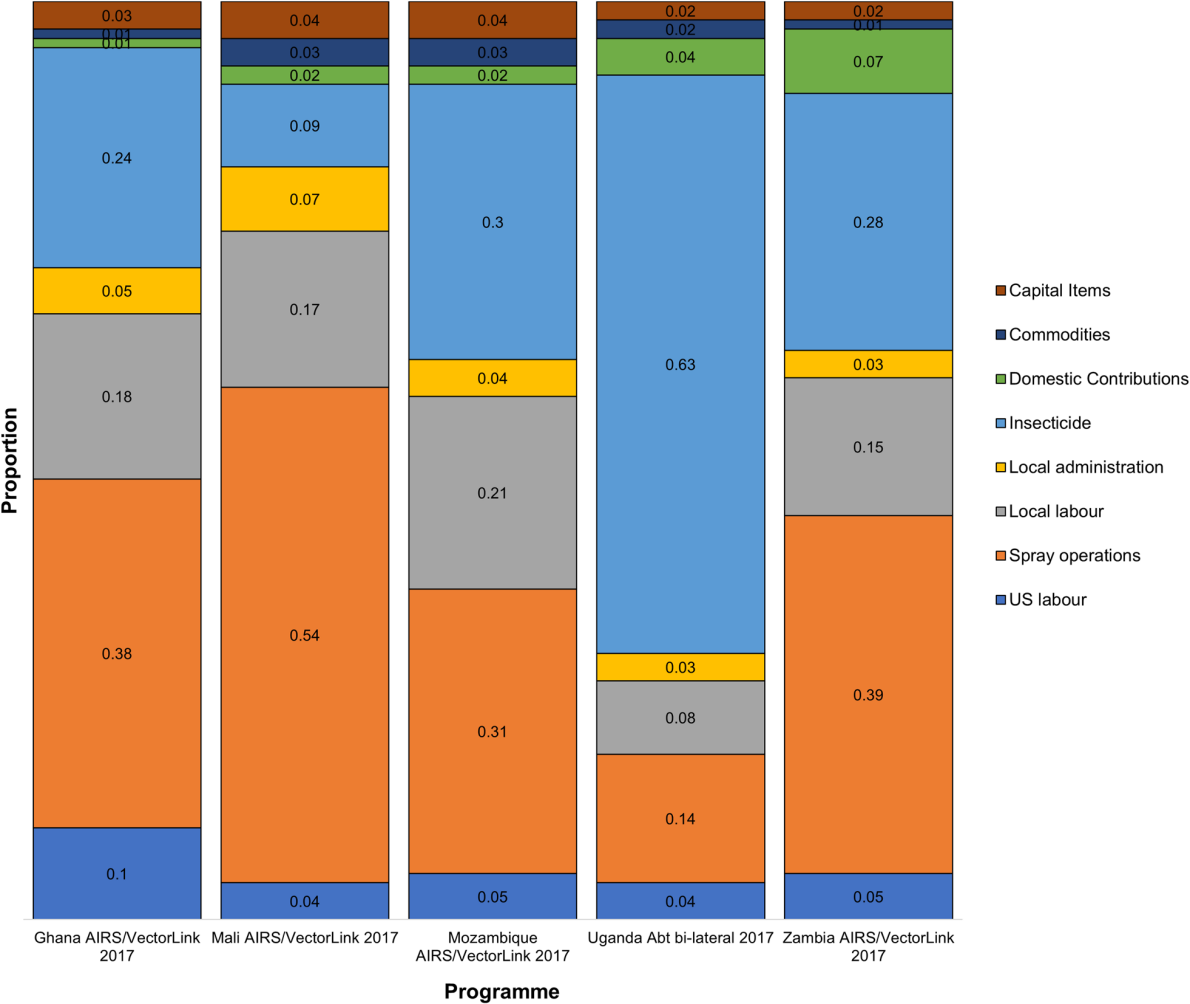
Contribution of line item expenses to total unit costs. AIRS, Africa Indoor Residual Spraying Project

The distribution of cost by line item in each programme (with the exception of AGAMal for which line items could not be easily harmonized with the other programmes) is shown in Fig. 2. These results indicate that across all programmes, the main cost drivers are insecticide, personnel, and operations (including transportation). The total cost per programme, the cost per person targeted, and the cost of the insecticide (per 100 m^2^) are presented in Table 4. The cost had a mean and standard deviation estimated as 5.33 USD per person targeted and 1.43 USD, respectively. The cost per person targeted was highest in the Mali programme and lowest in Zambia.

**Table 4:** Costs by programme

Programme	Year	Total cost (USD) (million)	Cost of insecticide (per 100 m^2^) (USD)	Total cost per person targeted (USD)
Ghana AIRS/VectorLink *Northeastern and Northern Regions*	2017	4.6	6.93	5.21
Ghana AGAMal *Upper West, Upper East Regions*	2017	6.4	6.10	5.42
Mali AIRS/VectorLink *Mopti Region*	2017	6.8	2.95	7.76
Mozambique AIRS/VectorLink *Zambezia Province*	2017	9.0	5.92	4.68
Uganda Abt bilateral *Northern and Eastern Regions*	2017	21.0	9.31	5.53
Zambia AIRS/VectorLink *Eastern, Luapula, Muchinga, Northern Provinces*	2017	10.0	7.04	3.35
*Mean*			*6.38*	*5.33*

AGAMal, AngloGold Ashanti Malaria Control; AIRS, Africa Indoor Residual Spraying Project; m, metre; USD, US dollars

### Impact and sensitivity analyses

The results of the base case scenario cost-effectiveness analyses are presented in Table 5. Overall, the results indicate that 3GIRS using Actellic^®^300CS is expected to be a cost-effective or highly cost-effective intervention in the sub-Saharan Africa context based on the results across the four specific IRS programmes chosen here.

**Table 5:** Incremental cost-effective ratio estimates for 3GIRS versus standard interventions

Programme	Year(s)	Cost per case averted	Cost per death averted (USD)	Cost per DALY averted (USD)	Cost-effectiveness estimate	Stringent highly cost-effective threshold (0.5 × GDP per capita)^a^ (USD)	WHO highly cost-effective threshold (GDP per capita)^a^ (USD)	WHO cost-effective threshold (3 × GDP per capita)^a^ (USD)
Ghana AIRS/VectorLink *Northeastern and Northern Regions*	2017–2018	3.20	1599	48	Highly cost-effective (by stringent standard)	1130	2260	6780
Mali AIRS/VectorLink *Mopti Region*	2017	6.76	3380	102	Highly cost-effective (by stringent standard)	467	933	2700
Uganda Abt bilateral *Northern and Eastern Regions*	2017–2018	41.25	20,624	625	Highly cost-effective (by WHO standard)	380	759	2277
Zambia AIRS/VectorLink *Eastern, Luapula, Muchinga, Northern Provinces*	2017	105.15	52,572	1593	Cost-effective	670	1340	4020

3GIRS, third-generation indoor residual spray; AIRS, Africa Indoor Residual Spraying Project; DALY, disability-adjusted life year; GDP, gross domestic product; USD, US dollars; WHO, World Health Organization

^a^GDP per capita extracted from International Monetary Fund’s World Economic Outlook

**Fig. 3:**
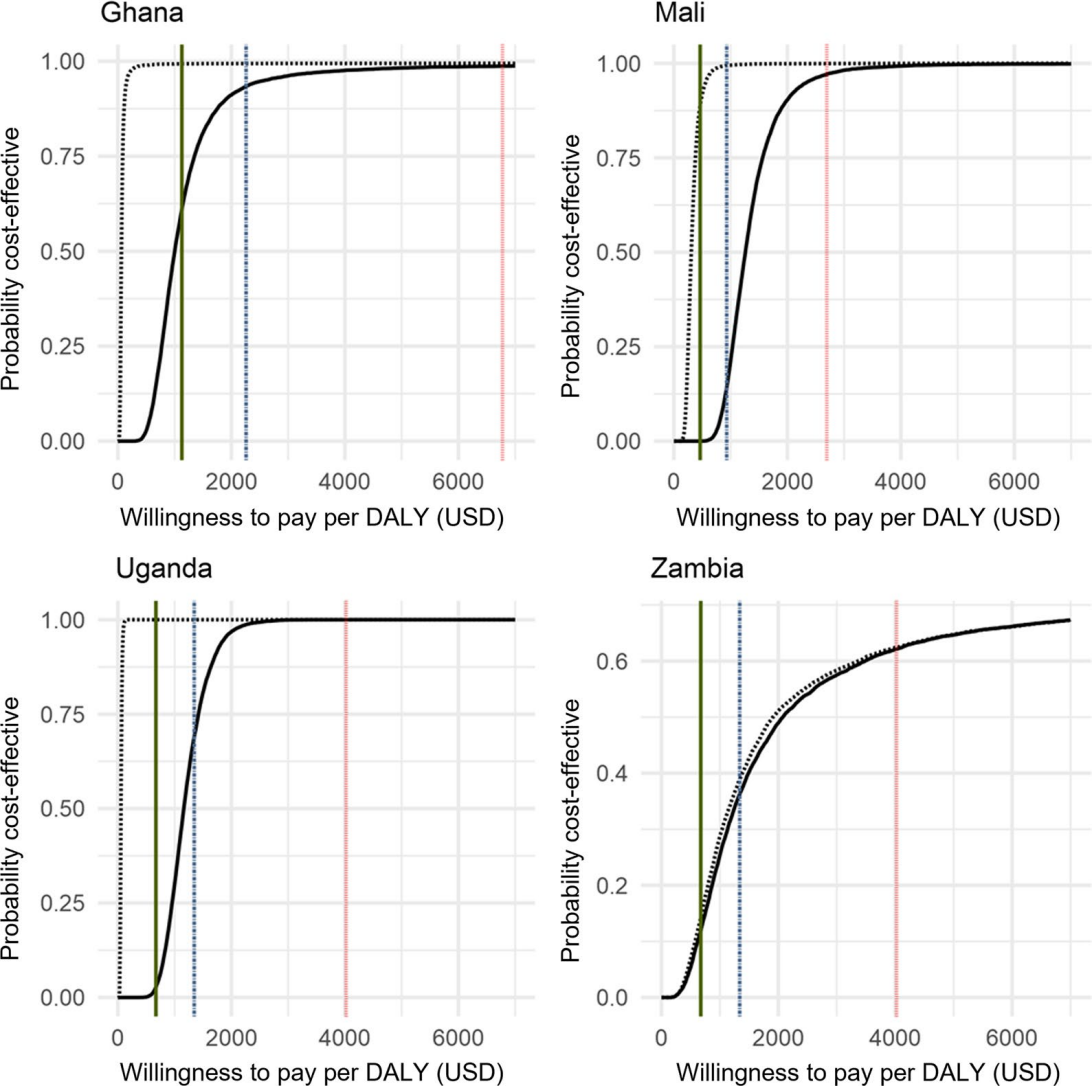
Cost-effectiveness acceptability curves for DALYs averted using 3GIRS in Ghana, Mali, Uganda, and Zambia. Vertical lines represent alternative cost-effectiveness thresholds: green solid line = 0.5 * per capita gross domestic product (PCGDP); dotted and dashed blue line represents 1 * PCGDP, and red dotted line represents 3 * PCGDP. Cost-effectiveness acceptability curves are represented with black curves: solid black represents a baseline incidence set at the national average incidence based on World Malaria Report data, dashed black represents baseline incidence set using study specific comparator district/health facility catchment incidence. 3GIRS, third-generation indoor residual spray; DALY, disability-adjusted life year; PCGDP, per capita gross domestic product; USD, US dollars

**Fig. 4:**
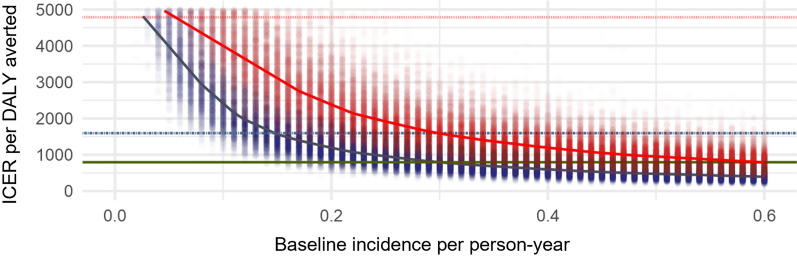
Global probabilistic sensitivity analysis results showing incremental cost-effectiveness ratio estimates for varied levels of incidence. Black points represent individual simulation results. Horizontal lines represent alternative cost-effectiveness thresholds: green solid line = 0.5 * per capita gross domestic product (PCGDP); dotted and dashed blue line represents 1 * PCGDP, and red dotted line represents 3 * PCGDP. The grey curve represents median ICER estimates at varied baseline incidence using the base case assumption of case fatality rate and red line represents median ICER estimates assuming a case fatality rate 50% lower than base case scenarios. DALY, disability-adjusted life year; ICER, incremental cost-effectiveness ratio; PCGDP, per capita gross domestic product

The country-specific probabilistic sensitivity analyses show significant heterogeneity with the variation in cost-effectiveness appearing to be driven largely by the expected baseline incidence. Overall, they demonstrate that 3GIRS using Actellic^®^300CS is an effective intervention across a range of transmission settings in sub-Saharan Africa and may be an attractive intervention to policymakers depending on local willingness to pay as shown by the cost-effectiveness acceptability curves in Fig. 3. The results indicate that 3GIRS using Actellic^®^300CS, would be considered highly likely (> 95%) to be a highly cost-effective intervention in Ghana, and a cost-effective intervention in Mali and Uganda even when national average malaria incidence was used as baseline. 3GIRS using Actellic^®^300CS is more likely than not (~ 55%) to be a cost-effective intervention in Zambia. When locally specific incidence rates were used as comparators, 3GIRS using Actellic^®^300CS was expected to be highly cost-effective in each of the Mali, Ghana, and Uganda sites even when based on the more stringent 0.5 times GDP per capita threshold. Results of the global probabilistic sensitivity analysis for various baseline incidence rates are shown in Fig. 4 and indicate that while assumptions about CFR are important to the underlying cost-effectiveness estimates, the baseline incidence in the location is a very important driver of heterogeneity. A one-way sensitivity analysis examines the incremental cost of 3GIRS compared to alternative products available at lower costs (Table 6).

**Table 6:** One-way sensitivity analysis of changing to a cheaper active ingredient

Programme	Year(s)	Unit cost per person targeted (USD)			
		Pirimiphos-methyl	Pyrethroid	Bendiocarb (1 × per year)	Bendiocarb (2 × per year)
Ghana AIRS/VectorLink *Northeastern and Northern Regions*	2017	5.21	4.17	4.38	7.96
Ghana AGAMal *Upper West, Upper East Regions*	2017	5.42	3.84	4.15	7.61
Mali AIRS/VectorLink *Mopti Region*	2017	7.76	7.17	7.29	13.18
Uganda Abt bilateral *Northern and Eastern Regions*	2017	5.53	2.63	3.21	6.01
Zambia AIRS/VectorLink *Eastern, Luapula, Muchinga, Northern Provinces*	2017	3.35	2.57	2.73	4.97

AIRS, Africa Indoor Residual Spraying Project; USD, US dollars

## Discussion

In each of the countries examined here, implementing IRS with Actellic^®^300CS, a third-generation product, as part of standard malaria control interventions (which also included mass distribution of standard LLINs) provided additional, cost-effective protection from malaria clinical incidence. Importantly, each of the IRS programmes evaluated here were implemented in (1) moderate- to high-burden regions (2) where universal coverage with standard LLINs was actively pursued through mass distribution campaigns and routine distribution channels, and (3) pyrethroid resistance was well documented in the local vector population. Collectively, results show that 3GIRS using Actellic^®^300CS deployed in addition to other standard interventions is expected to be a cost-effective, or even a highly cost-effective, use of public health resources across a wide variety of sub-Saharan African settings, despite the variation observed in the incremental costs per person targeted and per cases averted between country settings. The incremental costs calculated in this study did not consider the potential cost savings that might arise from reductions in treatment of uncomplicated and severe malaria or other indirect costs of malaria. They are, therefore, likely to be overestimates of the net cost of implementing these types of malaria control programmes.

The variations in the incremental costs likely result from several sources, including variation in the sprayed wall surfaces and house size relative to household populations, administrative and transportation and other necessary costs, the underlying malaria burden in the communities sprayed, and insecticide price. The cost of insecticide was one of the most significant cost drivers in nearly all programmes during the study period, contributing 9% to 63% of the total cost of the studied IRS programmes. Changes in product price can, therefore, make significant differences in the total programme cost. Switching to less expensive products may appear to be a cost-saving measure but using products with short effective lifetimes that require multiple spray rounds per year to maintain effectiveness (such as current carbamate IRS formulations) would result in large increases to overall cost. This is even the case assuming no additional management or capital costs would be required and only the spray operations and other similar recurrent costs would be doubled. Additionally, this assumes that the effectiveness of 3GIRS using Actellic^®^300CS could be achieved with these shorter-lived products. Ample evidence indicates that in situations where insecticide resistance to the older, less expensive products is common, the public health effect is not independent of the choice of active ingredient: more expensive products containing more efficacious and longer-lasting insecticide formulations are likely to be more cost-effective.

One major limitation of these studies is that in all cases, the use of 3GIRS with Actellic^®^300CS, in terms of effect and cost, is being compared to other standard interventions without any IRS. While this means that the comparison across countries is largely standardized, it also limits the direct comparison of these ICER estimates to be relevant to other supplemental malaria control interventions, such as larviciding or mass drug administration which are also applied in addition to LLINs. Further, the effectiveness or ICER for IRS in absence of LLINs cannot be reported, and these ICER estimates cannot be easily compared directly to LLIN estimates made with a comparator of no vector control. Nevertheless, this information could be used in future studies or to support modelling work, which would allow such comparisons and support decision-makers who design malaria control programmes. The effect and cost estimates in these studies rely on one 3GIRS product only, Actellic^®^300CS, potentially limiting their generalizability. Other 3GIRS products are expected to have similar effect profiles so long as the resistance profile where they are applied is similar; the costs of alternative 3GIRS products are also believed to be comparable. For these reasons, it is also expected that the cost-effectiveness estimates here, though entirely based on the deployment of pirimiphos-methyl in the form of Actellic^®^300CS, could be extrapolated to roughly correspond to other 3GIRS products.

The cost-effectiveness of 3GIRS clearly depends on context. In higher-incidence settings, a larger number of cases are expected to be averted for a similar expenditure on spraying, thus resulting in a much lower ICER estimate, as the absolute size of the effect in terms of cases, deaths, or DALYs averted increases while the absolute cost remains stable/fixed and is largely driven by target population size. Below an incidence of approximately 50 cases per 1000 persons per year, there is a low probability that IRS would be considered cost-effective in most sub-Saharan African settings by even the most lenient standards.

For example, the results from Zambia predict the intervention to be less cost-effective than in other settings in this study due mainly to lower baseline transmission intensity and despite relatively low costs per person targeted. Targeting IRS to higher-incidence areas would likely then lead to improved efficiency if the cost of targeting itself is not prohibitive and its accuracy is relatively high. Zambia uses subdistrict-level targeting partly for this reason. Such targeting to local areas of high incidence (through reactive approaches) has been shown to in some contexts to improve the cost-effectiveness of IRS [26].

Optimal intervention mixes depend not only on the cost-effectiveness of individual intervention strategies but also on overall programme goals, as well as an understanding of interactions between interventions and the cost-effectiveness of relevant alternatives. The ICER estimates show that the cost-effectiveness of 3GIRS with Actellic^®^300CS is expected to be in the range of other standard interventions. A systematic review published in 2011 found that the median incremental cost-effectiveness ratio per DALY averted ranged from 8.15 to 150.00 USD (~ 10.00 to 200.00 USD in 2020 value) for intermittent preventive treatment in pregnancy, ITNs, and IRS [7]. A similar review published in 2021 found a similar range in literature from 2005 to 2018 [8]. These systematic reviews generally covered studies where the comparator was no intervention; thus the ICER in the studies reviewed would be expected to be lower than when adding the intervention on to the other standard interventions, as was the case in this study since the ICER estimated in this study is effectively marginal rather than generalized. Even the estimates that are higher than the range from White et al. [7] in this case still fall well within the WHO threshold for consideration of an intervention as cost-effective in all cases. WHO thresholds, however, are not linked directly to empirical evidence of willingness to pay, nor are they directly derived from budget analysis. They are thus mainly useful as points of reference rather than as precise decision guides. The inclusion of a more stringent 0.5 GDP per capita threshold in the analysis also demonstrates the importance of willingness to pay in cost-effectiveness analysis and reflects the considerable debate about the specifics of such thresholds [23]. Ultimately, decisions to deploy an intervention, including 3GIRS, needs to be made in the context of the cost-effectiveness relative to available alternatives, affordability, and acceptability both politically and by local people, not based on generalized cost-effectiveness alone [27]. These thresholds here can mainly be used to determine, along with comparisons to other malaria control literature, that 3GIRS using Actellic^®^300CS, deployed in addition to other standard of care malaria control interventions provides acceptable value for money to be considered for use in a broad range of sub-Saharan African settings, including all those examined in this study.

The use of 3GIRS with Actellic^®^300CS in Ghana, Mali, and Uganda clearly showed larger effects in terms of cases averted than its application in Zambia. This is likely because the areas chosen for deployment of 3GIRS may have been specifically chosen due to their high malaria incidence and prevalence. This resulted in better (lower) ICER estimates from the field studies. This also indicates a more efficient use of resources than would have occurred had these programmes been deployed in lower-incidence areas of the countries. These results might not be maintained if the programmes were used in lower-incidence areas of the countries included in these studies.

A second major driver of the ICER estimate is the assumption chosen for the case fatality rate for community-acquired malaria infections [14]. If the rate used for these analyses is substantially higher than the true rate, then the ICER estimates presented here would be much more favourable in terms of cost per death and DALY averted. If the chosen rate is lower than actual, then the estimates presented here are conservative and biased against the use of 3GIRS. While there is considerable uncertainty, and almost certainly no single, broadly correct case fatality rate, sensitivity analysis showed that even reducing this rate by 50% would not alter the conclusion that 3GIRS with Actellic^®^300CS provides acceptable value for money in a broad range of sub-Saharan Africa malaria transmission contexts.

There are several additional limitations to the study approach used here. Differences in data quality and effect estimate calculations across study settings may result in challenges in pooling or comparing effect estimates. Additionally, the use of cross-country data to measure cost in IRS programmes may result in higher uncertainty in the estimate of the variability in cost than measures taken from within a single country over time. The estimates of variability in cost can then be thought of as conservative in the sense that variability is likely to be lower within a single programme than what was described in this study because price level differences across countries are likely more variable than changes over time in resource needs within countries. There is also substantial debate on the methods for calculation of DALYs and on the use of thresholds for determining cost-effectiveness. The approaches chosen here for calculation of DALYs (age-weighting and discounting) and the use of generalized cost-effectiveness thresholds are generally conservative, though the WHO thresholds of one and three times per capita have been suggested to be considerably higher than actual affordability and willingness to pay in many settings [28, 29]. Finally, the decision to ignore years of life lived with disability, means that the true burden of disease averted may be somewhat higher than estimated here.

The decision to estimate the gross ICER as opposed to net cost based ICERs likely means that ICERs from this study are overestimates compared to the real ICER. Additionally, the choice to use the provider perspective as opposed to societal and to exclude indirect benefits may also have effects in a similar direction. This is because cases of malaria averted may lead to cost savings to the health system and reduced incidence of malaria may lead to long-term benefits including increased human capital through varied capital formation mechanisms [30–32]. Unfortunately, the magnitude and mechanism for these cost savings or indirect benefit accruals are poorly understood and quantified. For example, marginal cost savings are likely not to be linearly related to cases averted because a significant portion of treatment costs are fixed [33, 34]. Indirect benefits may also accrue with high amounts of uncertainty and over a long period of time, well outside the time frame of this study. This study focused on gross costs which are likely to be higher than net costs and thus lead to a more conservative ICER estimate than if the ICER was based on net costs. Finally, while data on coverage and cost and effect of programmes are available, additional independent measures of programme quality are not; as such, some of the variations that appear in both effectiveness and cost may be driven by unmeasured variation in programme quality.

Current recommendations suggest that insecticides with different modes of action should be rotated or used in “mosaic” spraying to reduce the impact of insecticide resistance [35]. Switching of products could lead to substantial variation in budgetary needs year to year, and current and future 3GIRS products may also vary significantly in price, creating uncertainty in budget predictions. Use of multiple products in subnational rotational strategies could help to smooth such interannual variation in price as well as ensure the long-term cost effectiveness of 3GIRS as an intervention by helping mitigate the emergence of insecticide resistance. It is important to note that as the cost of newer products comes down through competition, market stabilization, and wider adoption of insecticide-resistance management plans that include product rotations, the cost-effectiveness of 3GIRS products is expected to improve. Ultimately, the choice to employ 3GIRS for spraying in any particular area will not depend on meeting WHO thresholds, but on individual country and donor programmes’ willingness to pay, the availability of financial resources to implement 3GIRS and the alternatives available in each setting.

## Conclusions

IRS with 3GIRS, using Actellic^®^300CS, is highly likely to be both an effective and cost-effective way to further reduce malaria burden when used in combination with other standard malaria control interventions in sub-Saharan Africa. Despite potential variations in both cost and effectiveness, there is substantial reason to expect that newer 3GIRS products will deliver cost-effectiveness at similar levels to the primary product studied here. Estimates of cost-effectiveness for 3GIRS using Actellic^®^300CS will vary substantially with the underlying malaria transmission context. How programmes can optimally combine 3GIRS delivery with other supplemental malaria control interventions—such as house improvement, larviciding, or chemoprevention approaches on top of standard malaria control interventions—will be highly context specific.

## Supplementary Information

**Additional file 1. **Description of malaria context and IRS programme implementation in Ghana, Mali, Mozambique, Uganda, and Zambia

**Additional file 2: Table S1. **Proportions of annual malaria burden falling within the 6-month IRS-analysis window, by country and year

## Data Availability

The costing datasets used and/or analysed during the current study are available from the corresponding author on reasonable request. Requests about the datasets on the impact of IRS should be directed to Molly Robertson, Senior Evidence Lead for the NgenIRS Project.

## Abbreviations

3GIRS: Third-generation indoor residual spraying
AGAMal: AngloGold Ashanti Malaria Control
AIRS: Africa Indoor Residual Spraying Project
CFR: Case fatality rate
CHEERS: Consolidated Health Economic Evaluation Reporting Standards
CI: Confidence interval
DALY: Disability-adjusted life year
DDT: Dichlorodiphenyltrichloroethane
GDP: Gross domestic product
ICER: Incremental cost-effectiveness ratio
IRR: Incidence rate ratio
IRS: Indoor residual spraying
LLIN: Long-lasting insecticidal net
NgenIRS: Next Generation Indoor Residual Sprays project
NMCP: National Malaria Control Programme
PMI: US President’s Malaria Initiative
USD: US dollars
WHO: World Health Organization
