# Supplementary material for: Incremental cost and cost-effectiveness of the addition of indoor residual spraying with pirimiphos-methyl in sub-Saharan Africa versus standard malaria control: results of data collection and analysis in the Next Generation Indoor Residual Sprays (NgenIRS) project, an economic-evaluation
Source: Malar J. 2022 Jun 11;21:185. doi: 10.1186/s12936-022-04160-3 (PMC9188086; doi:10.1186/s12936-022-04160-3)
Supplement: Supplementary file 1 — Additional file 1. Description of malaria context and IRS programme implementation in Ghana, Mali, Mozambique, Uganda, and Zambia [file 12936_2022_4160_MOESM1_ESM.docx]

# Ghana

The National Malaria Control Programme’s 2014–2020 Strategic Plan includes application of indoor residual spraying (IRS) in areas with high prevalence (greater than 40%).^1^ Costing data were collected on IRS implementation supported by the US President’s Malaria Initiative (PMI)–funded Africa Indoor Residual Spraying Project (AIRS)/VectorLink project led by Abt Associates (Abt) and AngloGold Ashanti Malaria Control (AGAMal) in 2017.

Malaria is endemic in Ghana, with seasonal transmission occurring during a six- to seven-month period in the north and a shorter three- to four-month period in the south.^1^ In 2017, *Anopheles gambiae* s.l. demonstrated high levels of resistance to pyrethroids but susceptibility to pirimiphos-methyl at PMI-monitored sentinel sites.^2^

## AIRS/VectorLink

In 2017, the AIRS project in Ghana conducted spray operations in seven districts: Bunkpurugu-Yunyoo, East Mamprusi, Gushegu, Karaga, Kumbungu, Mamprugu Moaduri, and West Mamprusi.^3^ In all spraying operations, the product Actellic®300CS was used. In the 2018 campaign, SumiShield® 50WG was used in addition to Actellic 300CS.

## AGAMal

In 2017, AGAMal conducted spray operations using Actellic 300CS in 13 districts: Obuasi, 9 districts in the Upper West region, and 3 districts in the Upper East region. In the 2018 campaign, 15 districts were sprayed using two active ingredients: Actellic 300CS and SumiShield 50WG.^1,4^

The Government of Ghana provided the policy and regulatory framework for effective grant implementation, including close working collaboration with key government institutions. The government also provide warehouse facilities in some districts, certification of insecticides, and supervision of environmental management activities.

# Mali

The Plan Stratégique de Lutte Contre Le Paludisme 2013–2017 includes universal insecticide-treated bednet coverage and blanket IRS coverage in high-risk districts.^5^ Costing data were collected on IRS implementation supported by the PMI-funded AIRS/VectorLink project led by Abt.

Malaria is endemic in the central and southern regions of Mali. In 2017, *An. coluzzi* was the predominant vector species observed at PMI entomological sentinel sites, and resistance or possible resistance to bendiocarb was observed in many locations.^6^ High levels of resistance to pyrethroids in *An. gambiae* were reported but to variable degrees depending on the location and the insecticide used.^7^

## AIRS/VectorLink

In 2017, the AIRS project in Mali conducted spray operations in the region of Mopti using Actellic 300CS, with involvement from Programme National de Lutte contre le Paludisme and the National Department of Sanitation. PMI has funded IRS in Mali since 2008 with the aim of reducing the malaria burden, especially among children under five years old and pregnant women. Due to observed short residual life of bendiocarb (two months), PMI switched to a long-lasting version of an organophosphate class insecticide (pirimiphos-methyl, Actellic 300CS) in two districts in 2014. In 2015, the programme switched to Actellic 300CS in all three districts: Koulikoro, Baroueli, and Fana.

# Mozambique

In Mozambique, the national vector control strategy prioritizes IRS coverage in areas with evidence of pyrethroid resistance but also considers malaria burden and population density.

Malaria is endemic in Mozambique. The country experiences year-round transmission that peaks in the April through December rainy season.^8^ In 2018, nationwide malaria prevalence was 39% in children aged 6 to 59 months and malaria was the leading cause of hospitalization and death in this vulnerable age group.^9^ Mozambique has a well-documented history of resistance to multiple pyrethroids (including, alpha-cypermethrin, permethrin, and deltamethrin) in local *An. gambiae* s.l. and *An. funestus* s.l. populations^10,11^ Costing data were collected on IRS implementation supported by the PMI-funded AIRS/VectorLink project led by Abt.

## AIRS/VectorLink

PMI supports the implementation of IRS in close collaboration with Mozambique’s National Malaria Control Programme (NMCP) and other governmental bodies at the provincial and district levels.^12^ Based on the vector susceptibility study data, with PMI approval and in partnership with the NMCP, AIRS Mozambique selected an organophosphate insecticide, Actellic 300CS. The project introduced it in 2015 and has used it as the insecticide of choice for all seven target districts since 2016.

# Uganda

Uganda experiences high levels of transmission throughout most of the country, with peaks in transmission coinciding with the two rainy seasons.^12^ Insecticide susceptibility testing in ten districts showed that *An. gambiae* was susceptible to pirimiphos-methyl in all tested districts tested and to bendiocarb in six of the districts. All districts showed resistance to pyrethroids.^13^ Costing data were collected on IRS implementation supported by a PMI-funded bi-lateral project led by Abt.

## PMI bi-lateral/VectorLink

In 2017, Uganda implemented IRS with support from PMI in nine high-burden districts in the Eastern region. With additional support from the United Kingdom’s Department for International Development, PMI supported IRS operations in an additional five districts in Eastern region.^12^ In 2018, the project sprayed 15 high-burden malaria districts in the northern (Alebtong, Amolatar, Dokolo, Lira, and Otuke) and eastern (Budaka, Bugiri, Butaleja, Butebo, Kaberamaido, Kibuku, Namutumba, Pallisa, Serere, and Tororo) parts of Uganda. District selection was based on the malaria burden, which was determined in collaboration with the Ministry of Health/NMCP and US Agency for International Development/PMI Uganda.^14^ The project used Actellic 300CS for the 2017 and 2018 spray campaigns in all 15 districts due to the insecticide’s long residual life, as documented in previous PMI-funded spray campaigns and data obtained from insecticide susceptibility tests conducted from 2012 to 2017.^14^

# Zambia

Zambia experiences year-round malaria transmission, with a peak in the November to April rainy season. *An. funestus* and *An. gambiae* are the main malaria vectors. Insecticide-resistance surveys in 2016 showed that both species are resistant to pyrethroids in Zambia.^15^ Costing data were collected on IRS implementation supported by the PMI-funded AIRS/VectorLink project led by Abt.

## AIRS/VectorLink

IRS is supported and implemented in Zambia by several different partners in different parts of the country. This analysis was limited to PMI-supported areas. PMI has supported IRS in Zambia since 2009. In 2017, the AIRS project in Zambia, in partnership with the Ministry of Health, conducted spray operations in 36 districts in Eastern, Muchinga, Northern, and Luapula provinces using the organophosphate insecticide, Actellic 300CS.

AIRS, in collaboration with Akros, used mobile devices for data collection and management (mSpray) in six districts in Eastern province (Nyimba, Katete, Chadiza, Vubwi, Mambwe, and Lundazi). All teams used standardized AIRS supervision and monitoring tools to assess the spray quality, environmental compliance activities, and spray data collection. AIRS collaborated with Dimagi to implement the Dimagi platform to ensure high-quality reporting and supervision in all 36 target districts. Following the spray campaign, the AIRS team conducted post-spray inventory activities and post-spray review meetings.

# References

1. US President’s Malaria Initiative (PMI). Ghana: malaria operational plan FY 2018. Bethesda, MD: PMI; 2018. <https://www.pmi.gov/docs/default-source/default-document-library/malaria-operational-plans/fy-2018/fy-2018-ghana-malaria-operational-plan.pdf?sfvrsn=5>. Accessed 24 Jan 2020].
2. PMI Africa Indoor Residual Spraying (AIRS) Project Indoor Residual Spraying (IRS 2) Task Order Six. Entomological monitoring of the PMI AIRS program in Northern Ghana: 2017 annual report. Rockville, MD: Abt Associates Inc.; 2018.
3. PMI Africa Africa Indoor Residual Spraying (AIRS) Project Indoor Residual Spraying (IRS 2) Task Order Six. Ghana end of spray report 2017. Bethesda, MD: Abt Associates Inc.; 2017. <https://www.pmi.gov/docs/default-source/default-document-library/implementing-partner-reports/ghana-end-of-spray-report-2017-indoor-residual-spray-irs-2-task-order-six-africa-irs-airs-project.pdf>. Accessed 30 Jan 2020.
4. AngloGold Ashanti Malaria Control Ltd (AGAMal). GF09 indoor residual spraying report: final performance after OSDV. Obuasi, Ghana: AGAMal; 2017. <https://agamal.org/wp-content/uploads/2018/04/GF09-Indoor-Residual-Spraying-Report_2017.pdf>. Accessed 30 Jan 2020.
5. Programme National de Lutte contre le Paludisme. Plan stratégique de lutte contre le paludisme 2013–2017. Bamako, Mali: Ministère de la Sante, République du Mali; 2013.
6. US President’s Malaria Initiative (PMI). Mali: malaria operational plan FY 2017. Bethesda, MD: PMI; 2017. <https://www.pmi.gov/docs/default-source/default-document-library/malaria-operational-plans/fy17/fy-2017-mali-malaria-operational-plan.pdf?sfvrsn=6>. Accessed 30 Jan 2020.
7. PMI Africa Indoor Residual Spraying (AIRS) Project. AIRS Mali entomological monitoring progress report. Bamako, Mali: Abt Associates Inc.; 2018.
8. US President’s Malaria Initiative (PMI). Mozambique: malaria operational plan FY 2018. Bethesda, MD: PMI; 2018. <https://www.pmi.gov/docs/default-source/default-document-library/malaria-operational-plans/fy-2018/fy-2018-mozambique-malaria-operational-plan.pdf?sfvrsn=5.>
9. Ministério da Saúde (MISAU), Instituto Nacional de Estatística (INE), ICF. Survey of Indicators on Immunization, Malaria and HIV/AIDS in Mozambique 2015. Maputo, Mozambique: MISAU, INE, ICF; 2018.
10. Abílio AP, Marrune P, de Deus N, Mbofana F, Muianga P, Kampango A. Bio-efficacy of new long-lasting insecticide-treated bed nets against *Anopheles funestus* and *Anopheles gambiae* from central and northern Mozambique. Malar J. 2015;14:352. <https://doi.org/10.1186/s12936-015-0885-y>.
11. PMI Africa Indoor Residual Spraying (AIRS) Project Task Order Six. Mozambique end of spray report 2017. Maputo, Mozambique: Abt Associates Inc; 2017. <https://www.pmi.gov/docs/default-source/default-document-library/implementing-partner-reports/mozambique-end-of-spray-report-2017-indoor-residual-spraying-(irs-2)-task-order-six-africa-irs-(airs)-project.pdf?sfvrsn=4>.
12. US President’s Malaria Initiative (PMI). Uganda: malaria operational plan FY 2018. Bethesda, MD: PMI; 2018. <https://www.pmi.gov/docs/default-source/default-document-library/malaria-operational-plans/fy-2018/fy-2018-uganda-malaria-operational-plan.pdf?sfvrsn=11>. Accessed 30 Jan 2020.
13. PMI VectorLink Project. Uganda annual entomology report, December 1, 2017–December 31, 2018. Rockville, MD: Abt Associates Inc.; 2019.
14. PMI VectorLink Project. Uganda 2018 end of spray report, April 9–September 18, 2018. Rockville, MD: PMI VectorLink Project, Abt Associates Inc.; 2018. <https://www.pmi.gov/docs/default-source/default-document-library/implementing-partner-reports/uganda-end-of-spray-report-2018-pmi-vectorlink.pdf?sfvrsn=4>.
15. US President’s Malaria Initiative (PMI). Zambia: malaria operational plan FY 2017. Bethesda, MD: PMI, 2017. <https://www.pmi.gov/docs/default-source/default-document-library/malaria-operational-plans/fy17/fy-2017-zambia-malaria-operational-plan.pdf?sfvrsn=7>. Accessed 30 Jan 2020.
