# Supplementary material for: Incremental cost and cost-effectiveness of the addition of indoor residual spraying with pirimiphos-methyl in sub-Saharan Africa versus standard malaria control: results of data collection and analysis in the Next Generation Indoor Residual Sprays (NgenIRS) project, an economic-evaluation
Source: Malar J. 2022 Jun 11;21:185. doi: 10.1186/s12936-022-04160-3 (PMC9188086; doi:10.1186/s12936-022-04160-3)
Supplement: Supplementary file 2 — Additional file 2: Table S1. Proportions of annual malaria burden falling within the 6-month IRS-analysis window, by country and year [file 12936_2022_4160_MOESM2_ESM.docx]

**Table S1. Proportions of annual malaria burden falling within the 6-month IRS-analysis window, by country and year**

|  | **Year 1** | | | **Year 2** | | | **Year 3** | | | **Average** |
| --- | --- | --- | --- | --- | --- | --- | --- | --- | --- | --- |
|  | Total incident cases over 12 months | Incident cases within 6-month IRS analysis window | Proportion of total annual burden analysed | Total incident cases over 12 months | Incident cases within 6-month IRS analysis window | Proportion of total annual burden analysed | Total incident cases over 12 months | Incident cases within 6-month IRS analysis window | Proportion of total annual burden analysed | Proportion of total annual burden analysed |
| **Mali** | 1360 | 1071 | 79% | 1254 | 922 | 74% | 1780 | 1297 | 73% | **75%** |
| **Ghana** | 3494 | 2339 | 67% | 3490 | 2400 | 69% | 5782 | 4338 | 75% | **70%** |
| **Uganda** | 5407 | 3795 | 70% | 2670 | 1955 | 73% | 2752 | 2157 | 78% | **74%** |
| **Average** | - | - | 72% | - | - | 70% | - | - | 75% | 73% |

Note: Cases represent the number of confirmed malaria cases (RDT + microscopy) per 10,000 person-months at risk in the no IRS comparator districts used in each analysis. Because the IRS campaigns are timed to be maximally effective during the highest-transmission months, approximately 75% of the expected annual case burdens fell within the 6-month post-IRS windows analysed.

IRS = indoor residual spraying; RDT = rapid diagnostic test.
